# Supplementary material for: Effects of Agronomic Practices on the Severity of Sweet Basil Downy Mildew (Peronospora belbahrii)
Source: Plants (Basel). 2021 Apr 30;10(5):907. doi: 10.3390/plants10050907 (PMC8147145; doi:10.3390/plants10050907)
Supplement: Supplementary file 1 [file plants-10-00907-s001.zip › plants-1191251-supplementary.pdf]

Table S1. Effect of tunnel orientation on the severity of sweet basil downy mildew (SBDM) in walk-in tunnels at Site 2.

**Experiment**	**Severity (%) [b]**			***Reduction (%) [d] of***	
	Time after planting (days)	East-West	North-South	*Severity values*	*AUDPC values (in Table 1)*
TD1	87	40.0±3.56 a[c]	35.6±2.12 a	-	*25.1*
TD2	87	50.6±7.45 a	34.8±3.5 b	*31.2*	*32.9*
TD3	98	95.0±7.42 a	11.3±3.24 b	*88.1*	
	112	98.0±1.42 a	80.0±2.26 b	*18.4*	*63.2*

[a] Experiments were carried out with sweet basil plants grown in sand in walk-in tunnels during the Spring 2014 (Experiments TD1 and TD2) and Winter 2014–2015 (Experiment TD3) growing seasons. Beds were covered with transparent polyethylene (TD1 and TD3) or left uncovered (TD2).
[b] SBDM severity was evaluated on a 0–100% scale, in which 0 = healthy plants and 100% = plants completely covered by SBDM symptoms/signs.
[c] Averages ± SE. Values in each pair followed by a different letter are significantly different according to one-way ANOVA with Tukey's HSD. Default significance levels were set at $\alpha = 0.05$.
[d] Disease reduction (%) was calculated according to the following formula: % disease reduction = 100 – 100 × (disease severity TT /disease severity control).

Table S2. Effect of increased air circulation on the severity of sweet basil downy mildew (SBDM) in greenhouses at Site 1a.

**Experiment**	**Severity (%) [b]**			***Reduction (%) [d] of***	
	**Time after planting (days)**	**Without fans**	**With fans**	*Severity values*	*AUDPC values (in Table 2)*
AC1	134	11.8±2.40 a[c]	7.3±2.00 b	*38.1*	*62.8*
AC2	69	2.3±0.45 a	1.0±0.48 b	*56.5*	*72.5*
AC3	109	35.4±3.57 a	11.2±3.26 b	*68.4*	*36.6*
	123	32.7±4.21 a	16.9±4.52 b	*48.3*	

[a] Field experiments were carried out with sweet basil plants grown in detached growth medium covered by gray polyethylene at Site 1 during the Autumn 2013, Spring 2014 and Winter 2015 seasons (Experiments AC1, AC2 and AC3, respectively).
[b] SBDM severity was evaluated on a 0–100% scale, in which 0 = healthy plants and 100% = plants completely covered by SBDM symptoms/signs.
[c] Averages ± SE. Values in each pair followed by a different letter are significantly different according to one-way ANOVA with Tukey's HSD. Default significance levels were set at $\alpha = 0.05$.
[d] Disease reduction (%) was calculated according to the following formula: % disease reduction = 100 – 100 × (disease severity TT /disease severity control).

Table S3. Effect of polyethylene mulch on the severity of sweet basil downy mildew (SBDM) and yield of sweet basil in greenhouses at Site 1a.

**Experiment**	**Severity (%) [b]**			***Reduction (%) [d] of***		***% yield increase (in Table 3)***
	Time after planting (days)	No mulch	Polyethylene	*Severity values*	*AUDPC values (in Table 3)*	
M2	114	28.1±2.31 a[c]	14.5±2.82 b	*48.4*	*51.0*	*6.4*
M4	93	35.1±5.43 a	23.8±4.36 b	*32.2*	*13.8*	*19.3*
M5	80	4.3±1.12a	1.5±0.68 b	*65.1*	*31.6*	-
	93	13.1±2.67a	9.6±3.19 a	-		
M6	123	53.1±5.34a	26.4±8.79 b	*50.3*	*64.1*	*42.3*
M7	109	50.0±2.95a	26.5±3.62 b	*47.0*	*47.4*	
	123	53.1±6.35a	26.4±7.55 b	*50.3*	*42.8*	*79.2*

[a] Field experiments were carried out with sweet basil plants grown in detached growth medium at Site 1 during the Autumn 2013 (M2), Spring 2014 (M4–M6) and Winter 2015 (M7) growing seasons. Beds were covered with gray polyethylene (M2, M4 and M5) or transparent polyethylene (M6 and M7).
[b] SBDM severity was evaluated on a 0–100% scale, in which 0 = healthy plants and 100% = plants completely covered by SBDM symptoms/signs.
[c] Averages ± SE. Values in each pair followed by a different letter are significantly different according to one-way ANOVA with Tukey's HSD. Default significance levels were set at $\alpha = 0.05$.
[d] Disease reduction (%) was calculated according to the following formula: % disease reduction = 100 – 100 × (disease severity TT /disease severity control).

Table S6. Effects of polyethylene mulch on the severity of sweet basil downy mildew (SBDM) and yield of sweet basil in walk-in tunnels at Site 2a.

Experiment	Severity (%) [b]			Reduction (%) [d] of		% yield increase (in Table 6)
	Time after planting (days)	No mulch	Polyethylene	*Severity values*	*AUDPC values (in Table 6)*	
M8	87	50.6±6.04 a[c]	24.5±4.03 b	*51.6*	*50.8*	-
M9	114	44.7±4.42 a	35.2±3.22 b	*21.6*	*40.2*	*13.2*
M10	114	71.3±4.72 a	47.2±9.95 b	*33.8*	*21.4*	*7.7*
M11	98	52.5±3.65 a	41.9±3.42 b	*20.2*	-	
M12	98	71.9±6.24 a	55.6±5.4 b	*22.7*	*17.3*	
M13	98	27.0±8.59 a	15.7±2.71 b	*41.9*	*26.5*	
	112	83.9±9.45 a	72.9±4.64 a	-		
M14	98	10.9±1.12 a	9.8±1.02 a	-	*48.1*	

[a] Experiments were carried out with sweet basil plants grown in detached growth medium at Site 2 during the Spring 2014 (M8–M10) and Winter 2014–2015 (M11–M14) growing seasons. Mulch-treatment plots were covered with transparent polyethylene.

[b] SBDM severity was evaluated using a 0–100% scale, in which 0 = healthy plants and 100% = plants completely covered by SBDM symptoms/signs.

[c] Averages ± SE. Values in each pair followed by a different letter are significantly different according to one-way ANOVA with Tukey's HSD. Default significance levels were set at $\alpha = 0.05$.

[d] Disease reduction (%) was calculated according to the following formula: % disease reduction = 100 – 100 × (disease severity TT /disease severity control).

Table S7. Effect of planting density (PD) on the severity of sweet basil downy mildew (SBDM) in greenhouses at Site 1a.

Experiment	Severity (%) [b]			Reduction (%) [d] of		
	Time after planting (days)	Dense (24)	Sparse (14)	*Severity values*	*AUDPC values (in Table 7)*	*Yield (in Table 7)*
PD1	114	50.6±6.22 a[c]	33.7±4.52 b	*33.4*	*41.5*	
PD2	114	7.9±1.53 a	2.5±0.71 b	*68.4*	*63.5*	*12.8*
PD3	134	5.6±1.27 a	2.1±1.20 b	*62.5*	*62.2*	
PD4	80	4.4±0.65 a	1.4±1.09 b	*68.2*	*22.1*	*31.5*
PD5	93	35.1±5.43 a	23.8±4.49 b	*32.2*	*33.9*	
PD6	151	10.0±1.68 a	6.0±1.61 b	*40.0*	*37.5*	*11.8*
PD7	151	22.9±2.25 a	14.7±1.14 b	*35.8*	*33.6*	

	Disease severity – Disease reduction [e]	
Equation		$y = 200281x^{-2.51}$
	r	0.9092
	n	7
	P	<0.01

[a] Experiments were carried out with sweet basil plants grown in detached growth medium in greenhouses at Site 1 during the Autumn 2013 (Experiments PD1–PD3), Spring 2014 (Experiments PD4–PD6) and Winter 2015 (Experiment PD7) growing seasons. Beds were left bare (Experiments PD1, PD4 and PD6) or covered with polyethylene (Experiments PD2, PD3, PD5, and PD7).

[b] SBDM severity was evaluated using a 0–100% scale, in which 0 = healthy plants and 100% = plants completely covered by SBDM symptoms.

[c] Averages ± SE. Values in each pair followed by a different letter are significantly different according to one-way ANOVA with Tukey's HSD. Default significance levels were set at $\alpha = 0.05$.

[d] Disease reduction (%) was calculated according to the following formula: % disease reduction = 100 – 100 × (disease severity TT /disease severity control).

e Equations for the relation between disease severity values of dense PD and significant disease reduction values by the sparse PD are presented and the Pearson regression value (r) is presented along with the significance levels (P).

Table S8. Effect of planting density (PD) on the severity of sweet basil downy mildew (SBDM) in walk-in tunnels at Site 2a.

**Experiment**	**SBDM severity (%) [b]**			***Reduction (%) [d] of***	
	**Time after planting (days)**	**Dense (30)**	**Sparse (15)**	*Severity values*	*AUDPC values (in Table 8)*
PD8	87	50.6±6.04 a [c]	44.2±5.12 a	-	*50.8*
PD9	72	27.0±3.94 a	16.5±4.23 b	*38.9*	*23.6*
	114	44.2±4.26 a	35.6±3.11 b	*19.5*	
PD10	72	18.4±3.42 a	10.7±2.42 b	*41.8*	*21.7*
	114	63.7±5.93 a	54.7±4.75 a	-	
PD11	98	16.0±5.24 a	7.6±2.41 b	*52.5*	*45.5*
PD12	98	78.8±5.42 a	50.0±4.47 b	*36.5*	*26.5*
PD13	112	83.9±2.58 a	72.9±3.42 b	*13.1*	*25.6*

**Disease severity – Disease reduction [e]**		
Equation		$y = 126.7e^{-0.037x}$
	r	0.7718
	n	6
	P	<0.10

[a] Experiments were carried out with sweet basil plants grown in sand in walk-in tunnels at Site 2 during the Spring 2014 (Experiments PD8–PD10) and Winter 2014–2015 (Experiments PD11–PD13) growing seasons. Beds were covered with polyethylene.

[b] SBDM severity was evaluated using a 0–100% scale, in which 0 = healthy plants and 100% = plants completely covered by SBDM symptoms/signs.

[c] Averages ± SE. Values in each pair followed by a different letter are significantly different according to one-way ANOVA with Tukey's HSD. Default significance levels were set at $\alpha = 0.05$.

[d] Disease reduction (%) was calculated according to the following formula: % disease reduction = 100 – 100 × (disease severity TT /disease severity control).

[d] Equations for the relation between disease severity values of dense PD and significant disease reduction values by the sparse PD are presented. Pearson regression value (r) is presented along with the significance levels (P).
